# Supplementary material for: Enhanced passive surveillance dengue infection among febrile children: Prevalence, co-infections and associated factors in Cameroon
Source: PLoS Negl Trop Dis. 2021 Apr 16;15(4):e0009316. doi: 10.1371/journal.pntd.0009316 (PMC8051767; doi:10.1371/journal.pntd.0009316)
Supplement: S1 Study Questionnaire — (PDF) [file pntd.0009316.s001.pdf]

STUDY QUESTIONNAIRE

Date : ……………………………   File N° ……………….… Tel:

I. IDENTIFICATION OF THE CHILD

1) Code …DENHIV xxxx.
2) Age ……….. Sex M ☐ F ☐
3) Residence…………..

I. DATA COLLECTION

1) Temperature………………..
2) Weight at birth (in grams) ………………………………….
3) Sleeps under mosquito bed net impregnated for long duration? Yes ☐ No ☐ (MILDA)
4) Consultation motives: ....
5) Do you know the HIV status of your child? Y ☐ N ☐
6) If No, would you like him/her to be tested? Y ☐ N ☐
7) If Yes, Positive? Negative?
8) If HIV positive, ARV Treatment: Y ☐ N ☐ If yes, since when? ....
9) Last CD4 count: ………… Specify the date: ………..
10) Does the child sleep under the impregnated bed net (MILDA): Y ☐ N ☐
11) Has your child ever been transfused Y ☐ N ☐

    If yes specify the number of times….

12) Any previous or current opportunistic infection (rash, mycosis, tuberculosis…) Y ☐ N ☐ If yes specify: …………
13) Does your child have fever? Y ☐ N ☐ If yes, since when……. …….
14) What is his/her temperature?

15) Auto-medication: Traditional medicine Y ☐ N ☐ Duration ……… Modern medicine Y ☐ N ☐

Duration ……………

16) Other signs or symptoms associated to the fever? Y ☐ N ☐ If yes specify

……………………………………………………..

17) Current body temperature taken by the nurse: ……………..

18) Are there water flasks around your compound? Y ☐ N ☐

19) Diagnosis upon admission: ………………………………….

II. SOCIODEMOGRAPHIC CHARACTERISTICS OF THE PARENTS (MOTHER)

1) Age:…………………………

2) Matrimonial Status: polygamy ☐ Monogamy ☐ widow ☐ Single ☐ Divorced ☐ Will not answer ☐

3) Profession: House Wife ☐ Public servant ☐ Agriculture ☐ Trader ☐ Liberal Profession ☐

other ☐ (specify) …………………

4) Ethnic group …………………………………………………….

5) Do you know your HIV status? Y ☐ N ☐ If yes, since when? …………….

If No, would you like to be tested?

III. RESULTS OF BIOLOGICAL ANALYSIS OF THE INFANT OR CHILD

1) Body temperature ……..
2) FBC……..
3) Blood smear: ………………………………
4) Malaria test: …………………………
5) Dengue test: …………………………
6) HIV test…………….
7) CD4+count: …………………………………
