# Supplementary material for: Enhanced passive surveillance dengue infection among febrile children: Prevalence, co-infections and associated factors in Cameroon
Source: PLoS Negl Trop Dis. 2021 Apr 16;15(4):e0009316. doi: 10.1371/journal.pntd.0009316 (PMC8051767; doi:10.1371/journal.pntd.0009316)
Supplement: Attachment — Submitted filename: Reply to reviewers.docx [file pntd.0009316.s002.docx]

Yaoundé, 23 April 2020

**To the editorial manager of Plos Neglected Tropical Diseases**

*Ref: PNTD-D-19-02118*

Reply to reviewers’ comments

Dear Editorial manager,

Thank you so much for the consideration of our manuscript, as well as the time taken by reviewers to provide constructive comments that will increase the clarity and understanding of our findings.

Below we are addressing all comments raised by the two reviewers. All modifications in the revised manuscript will be in red in the tracked version.

**Reviewer #1:**

This manuscript describes a cohort of acutely febrile children presenting to a single center in Yaounde, Cameroon, with the objective of describing incidence of dengue virus (DENV) infection, and associated co-infection with malaria and/or HIV among children with undifferentiated febrile illness. The authors describe the various tests used to detect DENV, malaria, and HIV. For DENV, the authors describe using the SD Bioline dengue Duo, an IgM/IgG ELISA, and PCR. However, it is not clear how they defined a case of DENV. For example, in line 92, the authors state that a positive DENV NS1 test result prompted confirmatory testing by PCR. Were samples that were NS1 positive but PCR negative not considered cases? What about discrepancies between NS1 and IgM results? This confusion is reflected later in the results.

This has been clarified in the revised manuscript. There was only one case of DENV NS1 positive that was confirmed by PCR. Also this DENV NS1 case was IgM positive as well.

The authors' use of the term prevalence throughout the manuscript is confusing. In the DENV literature, as well as that of most other infections, seroprevalence refers to presence of serum antibody, typically IgG, describing evidence of previous exposure/infection. The authors report anti-DENV IgG results as signifying previous infection, but the use of the term prevalence to describe both previous exposure and incident infection is confusing. Prevalence and incidence are not interchangeable.

You are correct. We have corrected the proper use of these two terms in the revised manuscript. The term prevalence has been changed to seroprevalence accordingly: IgM alone and IgM+IgG positive cases have been taken as reflecting the prevalence of dengue fever as the presence of IgM indicates early infection, wheras IgG was referred to as seroprevalence.

The presentation of the results is confusing. A diagram of how many subjects were classified into each of the diagnostic groupings would help to clarify the information provided in Table 2. Specifically, Table 2 indicates that there were 101 cases of DENV overall, with 70 cases positive for IgM. This implies that the remaining cases were due to NS1 positivity or PCR positivity. Yet, the text (lines 139-140) states that only 1 child was positive for NS1. A footnote in Table 3 explains that the 101 cases included both previous exposure and current DENV infections. The logic behind the grouping of previous exposure, signified by positive DENV IgG, together with incident infection, signified by positive DENV IgM, into a single group (referred to as "Ig dengue" in tables 3-5 but "Ag dengue" in tables 6-7?)  is neither intuitive nor explained. DENV IgG in combination with positive IgM might signify non-primary infection with a serotype that is heterologous to that of the previous infection. However this is not discussed and such heterologous non-primary DENV infections are not identified.

In order to provide clarifications and corrections, we have changed the presentation of our results as follow:

IgG positives represent previous exposure and is considered for seroprevalence.

IgM only represents new infections

All IgM positive represent current infections and is considered for the prevalence of infections.

All these categories have been explained in the text and in the tables.

As currently written, most of the results section simply restates information presented in the tables without providing additional interpretation. The tables can be presented in a more concise manner. There are seven tables, several of which (Tables 4 and 5 in particular) may be summarized in a sentence in the text or submitted as supplementary materials (see specific comments below).

As suggested, we have now presented some of the tables as supplementary materials. The results section has been restructured as you will see in the amended manuscript.

Since this manuscript is focused on DENV and associated co-infections, consider restructuring the results section to report DENV incidence first, then the co-infections in the context of DENV infection.

Thank you for this comment. We have restructured the results presentation in four main sections: Presentation of the population, Dengue infection, co-infections, associated risks

 The authors appropriately highlight the public health relevance that DENV infection occurs in children in Cameroon, frequently concurrently with either malaria and/or HIV, and that additional investigation of the effect of concurrent infection on disease presentation is warranted. However, discussion of associations between DENV and co-infection, which perhaps are the most interesting findings of the study, is superficial. Finally, the conclusion ends on speculation about disease severity, which is not addressed in this manuscript.

The discussion has been amended as you will see in the revised manuscript.

Specific comments:

1. Lines 54-55 are redundant

The sentence was modified by simply deleting the second half of the sentence.

2. Line 60: There is a licensed vaccine (Dengvaxia, Sanofi-Pasteur)

Thank you for the comment. The sentence was amended and now reads as follows: ‘No specific treatment exists to address the growing threat of dengue. But there is a licensed vaccine-Dengvaxia that could be used to prevent dengue infection.’

3. Lines 86-87: During which year(s) was the study performed? Also, the statement regarding transmission during the rainy months requires a reference.

The study was carried out in 2016.

We added three references to support the high transmission of dengue and plasmodium during the raining season; Polwiang et al, 2015 and Foo et al, 1985 and Odongo-Aginya et al 2005. ‘…rainy months with maximum transmission rate of both dengue and malaria, best for the breeding of their vectors (Anopheles and *Aedes*) as shown by other studies^16-18^.’

4. Consider providing the questionnaire in supplementary materials.

The questionnaire used to collect data has been added as supplementary material.

5. Line 92: Please specify a reference for the ELISA if this was an in-house assay, or the manufacturer if this was a commercial assay.

The test used was not an in- house, but a commercial kit. The manufacturer of the ELISA test used in this project was Standard Diagnostic, Inc and was added as suggested in the revised manuscript.

6. Line 99: Please clarify what is a "positive" urinary test?

To address this comment, we added as well the address of the manufacturer of the test used UriMed urinary test strips. A urinary test strip is recorded as positive by the presence of leucocytes and nitrites which are signs of urinary tract infection. This information was added in the revised manuscript.

7. Line 115: Stating the upper age range as 180 months followed by the mean in years is awkward. Consider referring the upper limit as 15 years.

So sorry! We amended as 15 years not 180 months.

8. Line 117: Please clarify what "living in an environment with stagnant water" means.

Living in an environment surrounded by water flasks. Read now in the revised version:’… and 46.9% were living in an environment surrounded by water flasks.’

9. Lines 128-129: 54 girls + 55 boys = 109 children. What about the other 74 children with malaria? Also Table 2 is referenced but does not include these data.

To address this comment, we have recalculated the proportions and it was meant to be: 93 girls and 90 boys. This is what can now be read in the revised tables. Also, we shifted the referencing just after the number of positive malaria cases.

10. Table 2: Dengue (Overall cases) is reported as 101, while DENV co-infections were 15 + 68 + 21 = 104. All DENV cases were co-infections? Please clarify.

Addressing this comment and taking into account all the other comments as well as for Reviewer 2, we redefined dengue cases as all the cases that were positive for IgM either with IgG or not (70 cases in total). So the data were revised as can be seen in the revised manuscript.

11. Table 2: Reporting of the CD4 count is poorly defined. Is this the mean or median? What does "Min: 240-400" mean? Consider reporting this in the text instead of in the table, since the remainder of the table reports frequencies.

Thank you for the suggestion. The CD4 data have been removed from revised table 2. In the text you will read: ‘For all participants, mean CD4 counts was 969 cells/mm3, normal for children aged 1 -15 years. The lowest CD4 count for HIV infected participants was 240 and that for dengue infected patients was 400.’

12. Lines 123-126 and table 2: Since this manuscript focuses on DENV, malaria, and HIV infection, consider reporting symptoms associated with these infections rather than the frequency of symptoms of the entire cohort.

We are now presenting the symptoms associated to dengue fever, malaria and HIV infection. No specific symptom was attributed to any of the co-infections. You will now read in the revised manuscript the following:’ Among children with positive blood smear, meaning confirmed malaria, the symptoms recorded were: fever, headache, sore muscle, anorexia, asthenia. For children with current dengue fever (IgM positive, alone or together with IgG) the following signs and symptoms were recorded: fever, headache, diarrhea, fatigue, vomiting, cough, rashes, lack of appetite). HIV positive children presented with fever, lack of appetite, fatigue and cough.’

13. Line 137: Is this the DENV case definition? What about the PCR testing referenced in the methods?

The PCR test was applied to the unique NS1 positive case we had during the study. And this case was also positive for IgM. This is why we did not make any reference to PCR test during the definition of dengue case in this work.

14. Table 4: Consider making this a supplementary table or excluding it altogether. The lack of association of any of the factors with sex can be concisely stated in the text. Additionally, the reference group should be identified when reporting odds ratios.

As suggested, table 4 will be presented as supplementary table in the revised manuscript and its contained data will be stated in the text.

15. Table 5: Please clarify the reference group for odds ratios. Is it the odds of having a particular test result for the specified age group over the odds for all other ages? Or is there a reference group? Consider making this a supplementary table or excluding it altogether since the lack of association with age can be stated in the text.

Table 5 will be provided as supplementary table and the results presented in this table stated in the text.

16. Lines 206-207: Please clarify the statement regarding susceptibility to infection and mother-to-child transmission.

In the revised manuscript, you will now read: ‘Our findings in a pediatric population are supported by reports showing more susceptibility of children to infection and by reported cases of mother-to-child transmission of dengue virus.’

17. Line 224: The principal targets of DENV infection are monocytes/macrophages/dendritic cells, whereas the principal targets of HIV infection are T lymphocytes. These are not the same cells.

To the best of my knowledge, HIV prefers T lymphocytes, but also target macrophages, monocytes and dendritic cells. All cells presenting CD4 on the surface are targeted by HIV. ( read webpath.med.utah.edu). For clarification, you will now read:…’especially for dengue and HIV as the two viruses all target immune cells, mainly T lymphocytes for HIV and both tissue macrophages, monocytes and dendritic cells’.

18. Line 231: Malaria and DENV are transmitted by mosquitoes, not flies.

Thank you. This has been changed and you will now read: ‘Malaria and DENV are transmitted by mosquitoes’

19. Lines 232-234: These statements are unsupported. DENV and malaria are transmitted by different mosquito vectors with different feeding habits (daytime vs. night time). Thus exposure is inherently different.

In order to address this comment, we have reformulated our sentence and you will now read: ‘Children living in this environment have the same risk to be exposed and infected with malaria (bitten by anopheles mainly during the night) and exposed and infected by dengue virus (bitten by *Aedes albopictus*/*aegypti* mainly during the day).

This manuscript reports the incidence of dengue virus (DENV) infection among children in Cameroon, along with associated co-infection with malaria and HIV. Given the paucity of data on DENV in Cameroon, the data offer an important contribution toward advancing knowledge of DENV in Africa. Unfortunately, this manuscript is disorganized and presents the data in a confusing fashion. The analyses are superficial and/or incomplete. These shortcomings detract from the potential impact of the study.

**Reviewer #2:**

The authors describe results of an enhanced passive surveillance study wherein children aged 6 months to 15 years presenting with undifferentiated fever were enrolled and tested simultaneously (I believe) for malaria, dengue, and HIV. The authors note that co-infections may be missed using standard testing practices. The results are quite interesting and could represent an important contribution to the literature regarding the clinical overlap between dengue, malaria, and HIV. However, the manuscript would benefit from increased precision in presenting results and discerning current and prior dengue infection based upon serological results.

MAJOR COMMENTS

-    Please place the study type in the abstract and title (presumably enhanced passive surveillance?).

We have addressed this comment and change the title of the revised manuscript as follow: ‘**ENHANCED PASSIVE SURVEILLANCE OF DENGUE INFECTION AMONG FEBRILE CHILDREN: PREVALENCE, CO-INFECTIONS AND ASSOCIATED FACTORS IN CAMEROON’**

-Provide further details about the methods and very basic descriptive statistics (number enrolled, age, etc,) in abstract.

We have now added the requested details in the abstract. Now in the revised abstract you will also read the following: ‘We carried out an enhanced passive surveillance and descriptive study during which 349 children aged 6 months to 15 years were enrolled’…

-    I don’t know that it is reasonable to conclude that DENV IgG-positive alone represents ‘dengue-infected’ – they may have had historical infection. I do agree that DENV IgM is more likely to be DENV (but may also represent ZIKV or some other cross-reactive flavivirus). I also agree that NS1-positive for DENV = acute infection. Similarly, for ‘co-infections’ I would not report that malaria smear + / DENV IgG + represents a co-infection.

To address this comment, our data were reviewed completely, defining dengue fever as positive IgM alone and IgM+IgG positive cases. Just to mention that the unique NS1 positive was also IgM positive. This refined definition of dengue positive has also been applied for co-infection cases. See revised manuscript.

-    Please be more precise in your presentation of findings – for example, in discussion line 209 – our findings showed a prevalence of 52.7% of malaria cases. You found that 52.7% of children presenting for care with acute undifferentiated fever had evidence of malaria sporozoites (correct?).

The above-mentioned sentence was amended and you will now read:’ Our findings showed that 52.7% of children presenting for care with acute undifferentiated fever had evidence of malaria sporozoites and 14.6% of them present with malaria-dengue co-infections.’

-    Please comment on how this testing algorithm differs from common practice in Cameroon (I’m guessing that malaria testing would be the first step, stop if positive?) and how your findings may indicate that an expanded approach may be needed.

To address this suggestion, in the revised discussion section, the following was added: ‘This study thus proposes a testing algorithm for fever cases. Instead of treating for malaria directly without a malaria test as it is the case (self-medication), the physician should propose both blood smear test or rapid malaria test as well as dengue test (DENV specific IgM/IgG rapid test), mainly because often patient present to the hospital not right away as symptoms present, but usually delay while trying to self-manage the infection.’

-    Relatedly, please comment on ‘next steps’ needed to better understand the clinical presentation and epidemiology of dengue and malaria / HIV coinfections in Cameroon. Your findings are very interesting and suggest that there may be opportunities to better understand how clinical severity and complications of dengue infection may be changed in the setting of co-infection.

Thank you so much for this constructive comment. To address this observation, and enhance our manuscript, the following was added: ‘Therefore, opportunities to better understand how clinical severity and complications of dengue infection and co-infection are also to be explored. Clinicians should pay more attention on the clinical presentation of their patients during treatment, have them recorded and analyzed, so as better treatment protocol be suggested to policies makers.’

MINOR COMMENTS

-    Please add ‘Cameroon’ and the general study type to the full title

This has been added as suggested. See revised title.

-    Introduction.

Line 60. There is in fact a licensed vaccine for dengue available in many countries (though not, to my knowledge, in Cameroon), however there are restrictions on the use given safety concerns.

This observation was also made by the first reviewer. It has been addressed as: ‘No specific treatment exists to address the growing threat of dengue. But there is a licensed vaccine-Dengvaxia that could be used to prevent dengue infection.’

-    Has there been an entomological survey done in Cameroon to document the geographic range of *Aedes aegypti* and *Aedes albopictus*? If so, would be good to mention. If not, would also be good to mention this knowledge gap.

Thank you for the comment. We are addressing it by adding the following paragraph:’ An entomological survey done by Simard et al in 2005 describes the circulation of both dengue vectors in Cameroon, *Aedes albopictus* and *Aedes aegypti* ^15^.’

-    Would refer to DENV ‘serotypes’ 1 and 2

This has been amended throughout the revised manuscript.

-    Please clarify that HIV, malaria, and dengue testing was performed for all individuals?

In the Material and Methods Section, we added a statement to correct this missing information: ‘The following tests were done for all included participants:’

-    Was there a time limit for the duration of fever? (I.e., less than 10 days?)

This information was collected from the questionnaire. The average fever duration was 4 days, with min been 1 day and maximum reported been 15 days. This information has been added in the first section of results.

-    Did you ask if the children slept under a bednet during the day? This is the biting period for Aedes mosquitoes

This specific information was not collected. The question that was asked was simply if the child sleeps under an impregnated mosquito’s bed net. Assuming that when a child is put to bed for sleeping he is protected with the bed net.

-    For table 1, the percents for stagnant water don’t add up to 100%

So sorry, the percentages were wrong. The recalculation gives 34.95% and 65.05% respectively for yes and No. See revised table 1.

-    Table 2: CD4 counts – is this the median?

Yes indeed. Mean CD4 count was 969 cells/mm3. As suggested by reviewer 1, this information was deleted in revised table 2 and reported just in the text.

-    Results, would change the ORs so that they reflect your description of results (e.g., line 155, ‘were less at risk of being infected’ – change OR to be (1/8.85)

-    Tables 6 and 7 seem to be mixed up in the text

-    Tables 6-7 – does ‘Ag dengue’ refer to ‘any immunoglobulin, IgM or IgG, to DENV’?

-    Table 6 – Does VIH refer to HIV?

Sorry, VIH is French. It was all replaced by HIV in the revised manuscript.

-    For all tables, footnotes defining abbreviations and the relevant diagnostic tests would be valuable

This was all amended accordingly.

-    It is unclear that is meant in line 206 about ‘findings are supported by reports showing more susceptibility to infection and cases of mother to child transmission of dengue virus’

This observation was also made by reviewer 1. It was addressed by reformulating the sentence as:’ Our findings in a pediatric population are supported by reports showing more susceptibility of children to infection and by reported cases of mother-to-child transmission of dengue virus.’

We hope to have addressed satisfactorily all comments.

We are really grateful for your time and inputs,

Sincerely,

On behalf of all co-authors

Céline Nguefeu NKENFOU
