# Supplementary material for: Enhanced passive surveillance dengue infection among febrile children: Prevalence, co-infections and associated factors in Cameroon
Source: PLoS Negl Trop Dis. 2021 Apr 16;15(4):e0009316. doi: 10.1371/journal.pntd.0009316 (PMC8051767; doi:10.1371/journal.pntd.0009316)
Supplement: Attachment — Submitted filename: Responses to Reviewers comments on R2 final.docx [file pntd.0009316.s004.docx]

January 11th, 2021

The Editor Plos Neglected Diseases,

Thank you very much for reviewing our manuscript "ENHANCED PASSIVE SURVEILLANCE OF DENGUE INFECTION AMONG FEBRILE CHILDREN: PREVALENCE, CO-INFECTIONS AND ASSOCIATED FACTORS IN CAMEROON" for consideration at PLOS Neglected Tropical Diseases.

Below, we are addressing the reviewers comments as requested.

In black ink the comments, in bleu and red the response to the comment.

Reviewer 2

MAJOR COMMENTS.

-    There remains some mixing of definitions of a dengue ‘case’ or dengue-infected individual. For example, in Table 2, a case seems to be defined as IgG or IgM (+). This is in contradiction to the primary aim of the study, which is to define ‘the prevalence’ (incidence?) of dengue in a cohort of children presenting with fever. IgG would reflect past infection (seroprevalence, not necessarily active infection) while IgM would reflect current or recent infection. Table 6, IgG antibodies are not necessarily suggestive dengue infection (as in the table heading) and it is confusing to consider them as such in the table.  For the purposes of the study, would define a dengue ‘case’ as IgM only and use these definitions more consistently.

-    Relatedly, please be consistent with your definition of ‘seroprevalence’ – Discussion, lines 196-197 – authors use seroprevalence to refer to both IgM and IgG. This is confusing and, again, reflects a mixing of definitions for acute and historical infection.

-    The authors may switch to using terms such as ‘the proportion of children presenting with fever who were found to have acute dengue infection was XX’ rather than ‘the prevalence of dengue’ – this is an acute infection (in contrast to malaria) and the over-reliance on the term throughout the manuscript to describe acute infections in the study is confusing when inter-mixed with references to seroprevalence.

All through the revision, we paid more attention to these definitions- seroprevalence, prevalence, active dengue, passed dengue infection…

INTRODUCTION:

-    Lines 53-54: recommend shortening to “dengue…is the most prevalent mosquito-borne viral disease worldwide and is described as a ‘neglected’….”

In the revised manuscript we removed the fragment: « neglected diseases although it is… »

-    Line 59-60. There is a licensed dengue vaccine available in many countries, but safety concerns and screening requirements limit its widespread use and impact.

We have added this information in the revised manuscript.

-    Line 65-66: A study of prevalence and distribution – looking at what population? Adults / children / all presenting with fever? This influences interpretation.

This was a substudy of arboviral epidemiological conducted during a large epidemiologic survey of HIV and other infectious diseases conducted by the Johns Hopkins Cameroon Program and the Cameroonian ministries of health and defense between 2000 and 2003. So their inclusion criteria did not mention fever.

-    Line 66: A study conducted in 2009 – what kind of study?

It was a cross sectional survey using a random cluster sampling method

-    Line 68: What type of climate? (Tropical? Subtropical?)

Tropical, Hot and humid

-    Line 72. Two serotypes identified (DENV-1 and DENV-2)

This was corrected as suggested

-    Lines 75-76 – would make very clear: the objective of the study was to determine…and associated risk factors in a pediatric population presenting with fever in Yaounde, Cameroon.

You will now read in the revised manuscript the following : « Therefore, the objective of this study was to determine the prevalence of dengue fever, dengue-malaria and dengue-HIV co-infections and associated risk factors in a pediatric population presenting with fever in Yaounde, Cameroon ».

METHODS:

-    Years of the study? Duration of the study? April to October of one year?

This study was carried out in 2015. We added this precision in the methodology section.

-    Any limits to the duration of fever for inclusion criteria? (i.e., within 7 days of onset of fever?)

No limit was set for the fever duration, as long as the main complaint for consultation was fever as declared by the parent/gardian.

-    Lines 102-103: Please refine sentence such as “Infants were enrolled only when their parent or guardian gave written proxy-consent” – but was this true only for infants? Assent obtained from older minors?

The sentence was refined as suggested.

The olders children aged above 5 gave their assent to be enrolled. This has been specified in the revised version.

-    Lines 134-138. Would move these definitions to the methods. Please rephrase “Were considered exposed” and “Were considered dengue infected” to be something like “Therefore, current or recent dengue infection was defined on the basis of a positive result for NS1 Ag and / or IgM.”

As suggested, we move the definition to the methods section.

RESULTS:

-    Clinical characterization: please provide information on the duration of fever, if known

Fever duration was unknown.

-    Line 123: “Highest temperature was 41”, “Average temperature was…”

Amended as : ”All children enrolled presented an axillary temperature higher than 37.5°C. Highest temperature was 41 ; lowest temperature was 37.8 and the average temperature was 38.9°C”

-    Line 123: Did you collect data only on active symptoms, or history of symptoms? For example, “12.6% reported a history of vomiting” versus “12.6% reported current symptoms of vomiting at the time of enrollment”

We collected current symptoms only.

-    Line 141.” The average CD4 count was 969 cells/mm3” “Children have a lower CD4 count were more likely to be positive”(Statistical test used, p value?)

To address this comment, we did the statistical analysis and amended to now read : ”Children having the lower CD4 count (240-400 CD4 cells/mm3) were more likely to be positve for malaria (20% compared to 10.8%; X2=0.71, p=0.4), HIV (40% compared to 0%; X2=31.08; p<0.001) or dengue (20% compared to 5.3%; X2 = 2.96, p=0.085) .”

-    Line 144. “The rate of Malaria – dengue coinfection was 19.5%”

This was amended as “ The rate of current dengue-malaria co-infection was 19.5% (68/349) and current dengue-HIV co-infection was 6% (21/349)”.

-    Line 145. “4.3% of the study population had evidence of current or recent dengue infection, as well as both malaria and HIV” (is this correct?

That is correct, the three studied infections althogether.

-    Line 152. What does “small raining season mean” – it was a low year for rainfall?

In the study site (Yaounde) there are four seasons : Two raining seasons and two dry seasons, one being longer than the other. It has been amended to « the shorter raining season »

-    Table 4. If the lowest age group is referent, why is the OR not 1?

The reference group is taken as the lowest age group. With your comment, we have converted this table as Figure 2.

-    Table 5. What is referent? Presumably, the absence of the other infection?

We added a note under table 5 to indicate this. Now table 4

-    Table 6. Presence of dengue IgG antibody is not consistent with your definition of ‘dengue infection’ rather ‘prior dengue exposure’. Please consider removing this comparison from the table – why would we think that historical exposure to dengue would be associated with diarrhea, for example?

You are correct, based on the definition of dengue infection, from table 6 (now table 5), we removed the lines concerning total immunoglobulines (Ig) and IgG ; leaving just IgM.

-    Lines 178-179. As above.

Agreed, to be consistent with the revised table 6 (now table 5).

DISCUSSION:

-    Lines 196-197. Confusing to use seroprevalence to refer to both IgM and IgG.

This was amended. We have used prevalence for NS1/IgM and seroprevalence for IgG.

-    Line 205. Please comment specifically on how your methodology may underestimate coinfection compared to the prior study.

In this line 205, we were presenting the fact that dengue malaria coinfection was higher is our study not the opposite, we had a proportion of 19% compare to 12.5% in the study of Monamele et al in 2018. Nevertheless, the explanation given was that the methodology used in the two studies for malaria diagnosis was different, we used microscopy, still considered as gold standard, while in the previous study the rapid diagnostic test was used. As well their population size of 33 malaria cases was smaller than our which was 183 cases. See revised manuscript.

-    Line 214. Recommend you remove the sentence beginning “In such setting”

This sentence was removed from revised version.

-    Line 216. “Viral coinfections” –Amended instead of Co-viral infections you will read “Viral coinfections”

-    Line 219. Capitalize Aedes and Anopheles -Done

-    Line 224-225. Aedes and Anopheles don’t exactly breed in the same conditions, but both may be increased during rainy seasons and thus may co-occur temporally

We have adjusted our sentence to reflect this comment. You will now read : « Malaria and dengue are transmitted by flies that multiply rapidly during rainy seasons and thus may co-occur temporally »

-    Line 226-227. Please refine final sentence, it is a little confusing

The last sentence was rephrased as follow :

“On the other side, HIV may be a risk factor for dengue and the reciproque may apply, given that both viruses target the same cells in the body”.

-    Please comment on the finding that sleeping under a bednet or living in an area without stagnant water was protective against dengue. These aren’t classically thought of as protective against Aedes – is this reflective of some other, related risk factor? (Socioeconomic status, etc?)

In order to clarify our statement, you will now read the following : «Our findings showed that children sleeping under bednet were less at risk from been infected by dengue and malaria, as bednet acts as shield and protects them from insect bites ».

-    You may consider commenting that the seroprevalence increased steadily with age (in fact, this may be a good figure to include, perhaps combining tables 3 and 4 to make space for a new figure) – this suggests that transmission is somewhat high and endemic (rather than epidemic, where we may be more even distribution across ages).

As suggested, we changed table 4 to figure 2. T he table presents some statistics, but not significant.. …

In order to comment on the effect of age on the seroprevalence, the paragraph below was added to the discussion : « Although no statistical significance was found between age and dengue Ab, the prevalence (IgM) and seroprevalence (IgG) increased steadily with age as shown in figure 2. This may be reflective of cumulative exposure, underlining the fact that dengue transmission is high and endemic rather than epidemic. As well as the child grows older, he become more independent in his movement and can pull away the bednet. »

We hope to have addressed all comments and that the reviewer will be satisfied.

Hope to hear back from you.

Best regards,

On behalf of all co-authors ,

Céline Nguefeu Nkenfou
