# Supplementary material for: Enhanced passive surveillance dengue infection among febrile children: Prevalence, co-infections and associated factors in Cameroon
Source: PLoS Negl Trop Dis. 2021 Apr 16;15(4):e0009316. doi: 10.1371/journal.pntd.0009316 (PMC8051767; doi:10.1371/journal.pntd.0009316)
Supplement: Attachment — Submitted filename: Responses to Reviewers comments.docx [file pntd.0009316.s005.docx]

March 02^nd^ , 2021

The Editor Plos Neglected Diseases,

Thank you very much for reviewing our manuscript entitled "ENHANCED PASSIVE SURVEILLANCE OF DENGUE INFECTION AMONG FEBRILE CHILDREN: PREVALENCE, CO-INFECTIONS AND ASSOCIATED FACTORS IN CAMEROON" for consideration at PLOS Neglected Tropical Diseases.

We thank the authors for their suggestions and constructive comments.

Below, we are addressing the reviewers comments as requested.

The comments are written in black ink, in bleu and red ink the response to the comment.

"Past" not "passed"

This was amended as suggested !

The following is still not a full sentence, please edit to a full sentence, for example “The highest temperature observed was…etc”: Highest temperature was 41°C, lowest  temperature was 37.8°C and the average temperature was 38.9°C.

This was amended as suggested !

You will now read : ‘’The highest temperature observed was 41°C, the lowest temperature observed was 37.8°C and the average temperature was 38.9°C.”

“Children having the lower CD4 count (240-400 CD4 cells/mm3) were more likely to be positve for malaria (20% compared to 10.8%; X2=0.71, p=0.4), HIV (40% compared to 0%; X2=31.08; p<0.001) or dengue (20% compared to 5.3%; X2 = 2.96, p=0.058) .” This only appears to be positive to HIV, so I would suggest rephrasing these results as it currently reads like you are suggesting that there are relationships with all HIV, dengue and malaria.

This comment was addressed and you will now read in the revised version :

Children having the lower CD4 count (240-400 CD4 cells/mm3) were more likely to be positve for malaria (20% compared to 10.8%; X2=0.71, p=0.4); for HIV (40% compared to 0%; X2=31.08; p<0.001) and for dengue (20% compared to 5.3%; X2 = 2.96, p=0.085) .

This refers just for children with lower CD4 counts.

“the reason being that they are less at risk of been bitten by flies”. This should be in the discussion, and the word flies needs to be replaced with either mosquito or vector. Similar for later reference to “flies”.

This was revised as suggested, and « flies » was replaced by «  mosquitoes »

“Stagnant water provide breeding conditions for Anopheles and Aedes.” This also should be in the discussion.

As suggested, this sentence was taken to the discussion section.

“IgG represents cases of previous exposure to dengue” I would suggest removing “cases of” here for clarity.

We have rephrased and you will now read : The presence of IgG is suggestive of previous exposure to dengue.

Discussion:

Please add to the comment about bednets being protective against dengue too, as bednets are generally thought to have less impact on dengue, due to the day biting of dengue transmitting mosquito.

To address this suggestion, you willl now read : « The reason being that they are less at risk of been bitten by mosquitoes, although bednets are generally thought to have less impact on dengue, due to the day biting of dengue transmitting mosquito ».

As the review suggested, could this also be a socio-economic factor or something else here?

Yes, indeed. As discussed in the paper, poor environmental hygiene can lead to the formation of stagnant water, which are breeding sites of mosquitoes.

“Among the children enrolled, 12.6% were HIV infected. Cameroon is said to be in a generalized HIV epidemic, although with decreasing prevalence, from 5.5 to 3.4% 21-23. Pediatric HIV is also a major public health problem although transmission is reduced due to the implementation of option B+ (Treat all HIV infected pregnant or breastfeeding mother despite CD4 count or viral load) for the prevention of 235 mother to child transmission 24. The percentage of HIV obtained in this study is in agreement with the national prevalence at the time of the study. Our study identified 21 out of 349 cases of dengue-HIV co infections, thus a prevalence of 6%.” I don’t understand the two % for HIV prevalence here- please rephrase.

The first prevalence is the overall prevalence and the second is the prevalence among children.

To clarify we rephrase and you will now read :  “The percentage of HIV obtained in this pediatric study population is in agreement with the national prevalence at the time of the study”.

“The result obtained in the present study is higher than the 9.8% previously reported by Demanou et al in Yaoundé among adults ^12^.” Please give more details on this study and which of the previous reported results from your study this is being compared to.

To address this comments the following paragraph was added to our revised manuscript : « This was a cross sectional survey conducted in three main cities in Cameroon (Douala, Garoua and Yaounde), using a random cluster sampling design  to document risk factors associated with anti-dengue virus Immunoglobulin G seropositivity in humans »

We hope to have addressed all comments !

On behalf of all authors,

Céline Nkenfou.
