# Supplementary material for: Enhanced passive surveillance dengue infection among febrile children: Prevalence, co-infections and associated factors in Cameroon
Source: PLoS Negl Trop Dis. 2021 Apr 16;15(4):e0009316. doi: 10.1371/journal.pntd.0009316 (PMC8051767; doi:10.1371/journal.pntd.0009316)
Supplement: Attachment — Submitted filename: Response to Reviewer comments.docx [file pntd.0009316.s003.docx]

**21 October 2020**
**To the editor: PLOS Neglected Tropical Diseases submission**

**Re: Manuscript No: PNTD-D-19-02118R1**

**ENHANCED PASSIVE SURVEILLANCE OF DENGUE INFECTION AMONG FEBRILE CHILDREN: PREVALENCE, CO-INFECTIONS AND ASSOCIATED FACTORS IN CAMEROON".**

**Response to reviewer comments**

We very much appreciate the reviewers’ and the editor’s comments concerning our manuscript and have addressed them as follows.
In addition to Reviewer 2 comments below, the AE has the following additional comments to help with the editing as suggested by Reviewer 2. This paper details an important study, but the analysis and the description of the results requires substantial editing.

**Reviewer #1 (Technical Comments to the Author):**

**Comment 1:** Abstract: Severe disease is generally refered to as severe dengue at the moment: <https://www.who.int/news-room/fact-sheets/detail/dengue-and-severe-dengue.>

“Dengue overall seroprevalence was 29.1%. Malaria cases were 52.7% and HIV cases represent 12.6%.” this line is unclear suggest rephrasing, seroprevalence suggests past infection, where as malaria and HIV are referred to as cases- please clarify what was being assessed.

**Response 1: The term seroprevalence has been changed to prevalence and the number changed from 29.1% to 20.2% which represents the prevalence of current or recent infection by Dengue.**

**Comment 2:** “But malaria either due to *P vivax* or *P falciparum* was associated to dengue infection, P=0.001 and 0.003 respectively. Likewise, HIV was significantly associated to dengue p=0.028.” It is unclear what is being assessed here- suggest rephrasing.

**Response 2: The whole sentence has been rephrased as requested.**

You will now read: “However, malaria as well as HIV were significantly associated with dengue (P=0.001 and 0.028 respectively).”

**Comment 3:** Author summary: “Dengue fever, transmitted by a mosquitoe as well as malaria is common among Cameroonian children.” Suggest rephrasing.

**Response 3: The sentence has been modified as requested.**

**You will now read: “**Dengue fever as well as malaria is common among Cameroonian children”

**Comment 4**: Introduction: “Both infections share similar clinical symptoms” requires reference.

**Response 4: Reference has been added as requested. Reference N°2**

There is now a vaccine for dengue- <https://www.who.int/immunization/diseases/dengue/revised_SAGE_recommendations_dengue_vaccines_apr2018/en/>

**Comment 6:** Results: P9 Line 137: Rephrase to a complete sentence.

Similar points throughout results, please ensure all sentences are complete sentences.

**Response 6: The results have been modified as requested to avoid making short phrases but complete sentences. See revised manuscript.**

**Comment 7:** What are the (%) in Table 3? This is currently very unclear, as is the interpretation from this table for when the peak transmission occurred. Much clarification needed.

**Response 7: The percentages in table 3 represent the occurrence of Dengue virus infection at different months of the year. They were calculated by dividing the number of infections for that month by the number of participants tested for that month. Below the table you can see that we have clearly defined the different categories: Ig which stands for immunoglobulin represents all cases of Dengue including both IgG and IgM.**

**We have replaced table 3 by figure 1 for better representation**

**Comment 8:** What are the (%) in Tables 5 and 6? This requires clarification/editing.

**Response 8: The percentages in Table 5 represent the prevalence of each infection type in each age group. This was calculated by dividing the number of positive cases by the number of samples tested for that specific age group.**

**Comment 9:** Line 182: I think the reference to table 6 and 7 are reversed. In addition, please provide further description of the co-infection relationship. At the moment in the text it is hard to understand what is meant.

**Response 9:** **Yes, indeed the references were reversed and we have now corrected. Further description of the co-infection relationships has also been provided as requested.**

**Comment 10:** I agree with reviewer 2, there needs to be further clarity on the past dengue infections and current dengue infections definition. In addition, how was a co-infection with dengue and the other pathogens defined? I think this should be current dengue infection, but it is not clear if this was how it was defined. This needs additional care and clarity for dengue, due to the acute nature of the infection and that the test used measure IgG and IgM (and clarity is needed on the interpretation of IgG positivity in this assay). Table 2 and the discussion of the results in the text needs to be clearer.

**Response 10: We have clarified the definition of current and past dengue infections in the test as requested. In fact, past infections are indicated by a positive IgG test and a negative IgM test while current infection in the case of the present study refers to the positive detection of IgM with a negative detection of IgG.**

**Comment 11**: Similarly, in the discussion what is meant by “prevalence” is this the % of febrile cases that are current dengue, or past dengue? I don’t think either is the “prevalence”. Please rephrase.

**Response 11: The term prevalence has been replaced by sero-prevalence.**

**Comment 12:** “Our findings are supported by reports showing more susceptibility to

infection and cases of mother to child transmission of dengue virus 16-17.” Please be clear which of your results are supported by this.

**Response 12: Precision has been made as requested.**

**You will now read: “**Our findings on the distribution of dengue are supported by reports showing more susceptibility to infection and cases of mother to child transmission of dengue virus ^16-17^.”

**Comment 13:** The dengue- HIV co-infection discussion requires clarification.

**Response 13: In the co-infections cases, acute dengue was used in the calculation. This has been clarified in the table (5) as foot note.**

**Comment 14:** “the common target cells”. Please state the target cells, and provide references.

**Response 14: The common target cells in this case refer to the CD4 cells. Reference has been provided as requested.**

You will now read in the revised manuscript: “Co-viral infections could even be worse especially for dengue and HIV as the two viruses share the common target cells such as CD4 positive cells ^20”^

Reviewer #2:

**Comment 1:** The manuscript has been improved from the prior submission; the authors’ thoughtful revisions are much appreciated. The discussion, in particular, is more solid. The classification of dengue illnesses as past and current remains a confusing aspect of the manuscript, and multiple small typological errors / areas for further clarification remain.

**Response 1: We appreciate the positive comment. A number of minor corrections have been made as well.**

**Comment 2:** General comments:

-    Not necessary to separate out IgM+ / IgG- and IgM+ / IgG+, this makes the presentation of results confusing. The timeline of specimen collection relative to onset of symptoms may influence whether IgM alone, IgM/IgG or IgG alone were detected but it is safe to say that detectable IgM+ is suggestive of acute / recent DENV infection. IgG+ alone suggests prior exposure. This simplification of presentation alone would clarify the abstract, results, table 2, and discussion.

**Response 2: The clarification on the definition of the type of infection has been made in the abstract and in the other parts of the text.**

**Comment 3:** Relatedly, please comment on the timing of specimen collection relative to the onset of symptoms (median and IQR). Perhaps add to table 1.

**Response 3: Samples were collected within 24hours of the onset of the symptoms. This has been clarified in the methodology section.**

**Comment 4:** The manuscript would benefit from a detailed review to correct typological errors and general flow of language.  This reviewer has pointed out a few specific areas to improve the flow of language below, but multiple other small typos remain. (e.g., abstract, line 40 ‘pic’ should be ‘peak’; Line 137, ‘with minimum been 1 day’ should be ‘with a minimum of 1 day’)

**Response 4: Corrections have been made as requested.**

**Comment 5:** Minor comments:

-    It is standard convention to define dengue virus as DENV, then use it throughout to refer to serotypes. ‘dengue’ is fine to use when describing dengue illnesses.

**Response 5: The term DENV has been replaced by dengue as suggested.**

**Comment 6:** The use of the term ‘prevalence’ is applied to very distinct concepts, i.e., % of febrile illnesses identified as dengue, seroprevalence. For percent of illness cases identified as dengue, perhaps move away from prevalence and describe as ‘the proportion of febrile illnesses identified as dengue’, for example.

**Response 6: The term prevalence has been replaced by sero-prevalence where needed as requested.**

Comment 7: Line 136 and Table 1. Water flasks or stagnant water? Does water flasks mean ‘containers’?

**Response 7: It is stagnant water. Stagnant water is part of the environment where water accumulate in holes and sometimes create mud.**

Comment 8: Lines 142-146. For each of these infections, are symptoms reported for single infections (i.e., HIV alone, no dengue or malaria) or for a given infection regardless of coinfections? Consider a supplementary table showing symptoms for each virus alone, coinfections, comparing severity and clinical presentation.

**Response 8: These are participants who presented at the clinic with fever. The objective is to determine the sero-prevalence of Dengue and it’s co-occurrence with malaria and HIV.**

Comment 9: Lines 166-167 is very confusing. Hopefully can be simplified as in first general comment above.

**Response 9: More clarifications have been provided as requested.**

Comment 10: Table 2. Please be explicit in table about the fact that you are considering acute dengue (not prior dengue) in coinfection estimates (as you have added to the text).

**Response 10: This information has been added as requested.**

Comment 11: Table 3 – perhaps turn into a figure showing percents by month?

**Response 11: The table has been changed into a figure as suggested.**

Comment 12: Supplemental table 4. Please indicate what is the referent group for the odds ratios (i.e., are the odds of IgG lower in females?)

**Response 12: The reference group has been indicated as requested.**

**“**Male is considered as the reference”

Comment 13: Supplemental table 4. Please indicate what is the referent group for age the odds ratios i.e., is it age 2-5 years compared to all other age groups? You may consider making 0-2 the referent group for subsequent age groups)

**Response 13: The reference group has been indicated as requested.**

**“**The lowest age group was considered as the reference”

Comment 14: Line 290. This sentence about viral infections being more severe in children could be softened. Chickenpox, for example, is more severe in adults.

**Response 14: The sentence has been softened as requested.**

You will now read “…especially in infants where both viral diseases are sometimes more severe”

We hope we have satisfactorily addressed all comments

Sincerely

On behalf of all co authors

Prof Céline Nguefeu Nkenfou
